# School Provision of Universal Free Meals and Blood Pressure Outcomes Among Youths

**DOI:** 10.1001/jamanetworkopen.2025.33186

**Published:** 2025-09-25

**Authors:** Anna M. Localio, Paul L. Hebert, Melissa A. Knox, Wyatt P. Bensken, Aileen M. Ochoa, Jennifer Sonney, Jessica C. Jones-Smith

**Affiliations:** 1 Department of Health Systems and Population Health, University of Washington School of Public Health, Hans Rosling Center for Population Health, Seattle; 2 Health Services Research & Development Center of Innovation for Veteran-Centered and Value-Driven Care, Veterans Affairs Puget Sound Health Care System, Seattle, Washington; 3 Department of Economics, University of Washington, Seattle; 4 OCHIN, Inc, Portland, Oregon; 5 Department of Child, Family, and Population Health Nursing, University of Washington School of Nursing, Seattle; 6 Department of Health, Society and Behavior, Joe C. Wen School of Population and Public Health, University of California, Irvine

## Abstract

**Importance:**

The Community Eligibility Provision is a federal universal free school meals policy for schools in low-income areas. Expanding access to school meals, which are children’s most nutritious food source, may be a health-promoting policy.

**Objective:**

To assess whether school-level adoption of the Community Eligibility Provision was associated with childhood blood pressure outcomes.

**Design, Setting, and Participants:**

This cohort study used a difference-in-differences design for staggered policy adoption, observing low-income public and charter schools in 12 US states longitudinally from 2013 through 2019. The medical records for patients aged 4 to 18 years receiving care from community health organizations in the OCHIN health care network were matched to schools based on address. Data were analyzed from April 1 to July 5, 2024.

**Exposure:**

School participation in the Community Eligibility Provision.

**Main Outcomes and Measures:**

The primary outcome was the annual school-level proportion of patients with a high blood pressure measurement (at or above the 90th percentile for age, sex, and height), and the secondary outcomes included a hypertensive measurement (at or above the 95th percentile) and mean systolic and diastolic blood pressure percentiles.

**Results:**

The sample included 1052 schools matched to 155 778 distinct patients. The mean (SD) proportions of patients based on race and ethnicity were as follows: 0.04 (0.08) Asian patients, 0.46 (0.33) Hispanic patients, 0.01 (0.03) patients of multiple races, 0.01 (0.02) Native Hawaiian or Other Pacific Islander patients, 0.13 (0.22) non-Hispanic Black patients, 0.25 (0.26) non-Hispanic White patients, and 0.09 (0.09) patients with unknown race and ethnicity. The majority of schools (n = 670 [63.7%]) were located in California or Oregon. School participation in the Community Eligibility Provision was associated with a −2.71 percentage point (95% CI, −5.10 to −0.31 percentage point; *P* = .03) net reduction in the proportion of patients with a high blood pressure measurement, corresponding to a −10.8% (95% CI, −20.4% to −1.2%) net decrease over 5 years. Participation was also negatively associated with the proportion of patients with a hypertensive measurement and with the mean diastolic blood pressure.

**Conclusions and Relevance:**

This cohort study of schools matched to child and adolescent patient medical records from a large network of community health organizations found that school participation in the Community Eligibility Provision was associated with a net reduction in blood pressure outcomes. These findings add to mounting evidence that universal free school meals may be associated with improved child health.

## Key Points

**Question:**

Is school participation in the Community Eligibility Provision, a universal free school meals policy, associated with childhood blood pressure outcomes?

**Findings:**

In this cohort study of 1052 schools matched to medical records from 155 778 patients receiving care from community health organizations, Community Eligibility Provision participation was associated with an 11% net reduction over 5 years in proportion of patients with a high blood pressure measurement.

**Meaning:**

These findings suggest that universal free school meals policies such as the Community Eligibility Provision may be associated with improved population health.

## Teaser

This cohort study assesses whether school-level adoption of the Community Eligibility Provision was associated with childhood blood pressure outcomes among students in low-income public and charter schools in 12 US states.

## Introduction

Pediatric high blood pressure (BP) increases the risk of hypertension in early adulthood, a leading cause of cardiovascular and kidney disease.^1,2,3,4,5^ High BP, encompassing elevated BP (formerly referred to as *prehypertension*) and hypertension, is defined as systolic or diastolic BP at or above the 90th percentile for age, sex, and height based on children with normal weight.^1^ The prevalence among youths is estimated at 11% but increases with age and adiposity.^1,2,6^ Causes include obesity, unhealthy diet, and sedentary lifestyle,^1,7^ which are influenced by fundamental causes of disease such as socioeconomic status and structural racism.^8,9^ As such, high BP disproportionately burdens children from low-income and racially and ethnically minoritized populations, particularly Black and Hispanic children.^1,6^ Disparities persist even after adjusting for weight status.^6^

A recommended nonpharmacological treatment of high BP is the Dietary Approaches to Stop Hypertension diet, which is high in fruits, vegetables, and low-fat dairy and low in sodium and saturated and trans fats.^1^ However, there is currently no public health mechanism for disseminating this diet and ensuring widespread adoption. Revised standards to meals served through the National School Lunch Program and School Breakfast Program, however, have led to school meals that more closely resemble the Dietary Approaches to Stop Hypertension diet. The 2010 Healthy, Hunger-Free Kids Act enacted legislation requiring schools participating in these programs to serve meals aligned with the Dietary Guidelines for Americans,^10,11^ These stricter standards led to the improved nutritional quality of school meals,^12,13,14^ which are healthier than alternatives and are children’s most nutritious food source.^15,16^

Nearly all public and charter schools participate in the National School Lunch Program and School Breakfast Program, administered through the US Department of Agriculture. These programs provide free and reduced-price meals to children from income-eligible households. Although more than 28 million children participate in the National School Lunch Program and more than 14 million children participate in the School Breakfast Program,^17^ school meals are not accessed by all who could benefit. Many households above the income eligibility threshold for free or reduced-price meals ($57 720 annually for a family of 4)^18^ struggle to afford full-price school meals.^19^ Additionally, stigma may prevent income-eligible households from participating.^20,21^ Policy initiatives to improve school meal access could reduce the risk of high BP in children.

The Community Eligibility Provision (CEP), also authorized by the Healthy, Hunger-Free Kids Act, is a universal free meals policy, allowing schools with a high proportion of students from low-income households to provide free breakfast and lunch to all students. CEP increases school meal participation and may improve diet quality by substituting up to half of students’ diets with healthier options.^22^ CEP became available nationwide in 2014; since then, more schools have adopted the policy each year. In 2024, more than 47 000 schools participated, reaching more than 23 million children.^23^ Additionally, after the US Department of Agriculture temporarily issued pandemic waivers allowing all schools to offer universal free meals, several states have implemented their own policies, supplementing federal funding with state funds. Given the popularity of such policies, understanding their health effects is critical. CEP has the potential to improve population health, as supported by emerging evidence showing associations with obesity reduction.^24,25,26^ CEP may reduce high BP, not only through improved nutrition and obesity reduction, but also through reduced household income constraints and food insecurity.^22,27,28,29^ This study assessed whether attending a CEP-participating school was associated with BP outcomes.

## Methods

### Overview

This cohort study employed a difference-in-differences design for staggered policy adoption. We used electronic health record (EHR) data from patients aged 4 to 18 years who attended community health organizations in several US states between 2013 and 2019. The University of Washington institutional review board determined the research exempt from oversight and from informed consent because the research posed no more than minimal risk to patients’ identification. The study is reported in accordance with the Strengthening the Reporting of Observational Studies in Epidemiology (STROBE) reporting guideline.

### Inclusion Criteria

Patient data were obtained from the OCHIN Epic EHR system; OCHIN is a national nonprofit health information technology consultancy that provides a fully hosted and shared EHR platform for a nationwide network of community health organizations.^30^ Patients in kindergarten through 12th grade who visited a clinic in the OCHIN network during the school year (August 15 through June 30) between 2013 and 2019 were matched to their neighborhood school based on address at the time of visit. If patients were matched to multiple schools, we chose the school closest to their address. If a patient moved during the study, they were reassigned to a new school.

eFigure 1 in Supplement 1 depicts a flowchart of sample exclusion criteria. Beginning with 11 534 schools, we excluded 6774 schools that were ineligible for CEP between 2014 (when CEP became available nationwide) and 2019. Schools were considered eligible for CEP if they had an identified student percentage (ISP) of 40% or greater. The ISP is determined by the proportion of students directly certified for free meals through household participation in a federal assistance program. The ISP was obtained from state departments of education and the Food Research & Action Center.

We excluded 94 schools without patient observations during the study period and 117 schools that matched only to patients with missing or implausible height or BP values. We used 2 algorithms to identify implausible measurements: 1 for cleaning child growth data from EHR data (eg, extreme values, duplicates, and carried forward)^31^ and 1 for computing BP percentiles based on Centers for Disease Control and Prevention growth charts and normative BP tables.^1,32^ To ensure that results are not due to schools entering or leaving the sample, we excluded 3309 schools without patient observations in all school years 2013-2014 through 2018-2019. Most of these excluded schools (75%) did not have multiple observation years or an untreated reference year (required for our analytic approach). To obtain reliable school-level outcome measurements, we excluded 184 schools with an average of fewer than 5 patient measurements per year. Finally, we excluded 2 schools that participated in a pilot version of CEP and 2 schools with missing CEP participation status.

### Outcomes

The unit of analysis was the school year. The primary outcome was the proportion of OCHIN patients with a high BP measurement, defined as a systolic or diastolic BP measurement at or above the 90th percentile based on age, sex, and height for children with normal weight (defined as body mass index [BMI] below the 85th percentile for age and sex based on Centers for Disease Control and Prevention growth charts).^33^ Secondary outcomes included the proportion of patients with a hypertensive BP measurement (at or above the 95th percentile) and the mean systolic and diastolic BP percentiles with 95% CIs. If a patient had multiple visits, we used the visit nearest to June 30 to contribute to the outcome calculation. If multiple measurements were taken within a visit, we used the mean value to contribute to the outcome calculation. We aggregated BP outcomes at the school level because this was required for our analytic approach and because BP measurements are highly variable at the child level.

### Exposure

Schools were considered treated if they participated in CEP. Participation data were obtained from state departments of education, the National Center for Education Statistics Common Core of Data,^34^ and replication data from another researcher.^35^

### Covariates

Models included the time-varying patient characteristics by school and year that may cause both selection into a CEP-participating school and BP outcomes (but were not hypothesized to be on the causal pathway), to account for compositional changes in patient-level demographics between years. Characteristics included mean age, mean proportion of male patients, mean proportion of patients with public health insurance, and mean proportion of patients based on the three largest race and ethnicity groups (Black, Hispanic, and White, which made up the majority [84%] of patients in the sample). Race and ethnicity were self-reported by patients in the EHR. We also included state Medicaid expansion status as a time-varying covariate.

### Statistical Analysis

We computed summary statistics (mean [SD] values, weighted by mean number of patients per school, or number and percentage) by patient and school characteristics overall and by year of first policy adoption. To assess the association of CEP participation with BP outcomes, we used a difference-in-differences approach for staggered policy adoption, allowing for heterogeneous treatment effects over time. This approach, described by Callaway and Sant’Anna,^36^ computes weighted difference-in-differences estimates for each cohort (year schools adopt the policy) and school year, controlling for all observed and unobserved time-invariant, school-level confounders. *P* < .10 was used as a threshold for statistical significance, consistent with other studies on CEP.^24,25,35^

The analysis included 5 cohorts: schools that adopted in school years 2014-2015, 2015-2016, 2016-2017, 2017-2018, and 2018-2019. Eligible schools that did not adopt CEP during the study period were also included. For each cohort, the difference in outcomes between each year and the reference year (1 year prior to policy adoption) was compared with the same difference in outcomes for a control group of not-yet-treated schools (including both schools that adopted in later cohorts and those that did not adopt during the study period). Estimates were aggregated into an overall treatment effect estimate, as well as by cohort and by years since policy adoption. This approach avoids the potential biases of applying traditional 2-way fixed-effects models (linear regression models that include unit and time fixed effects) to settings with a staggered policy adoption.^37^

Models used doubly robust estimation based on stabilized inverse probability of treatment weighting and generalized least-squares regression.^36,38^ The inverse probability of treatment weighting model uses propensity scores to derive inverse probability weights using stabilized weights to reduce extreme values and prevent very large weights from having undue influence.^38,39^ If either the inverse probability of treatment weighting or the outcome regression model (but not both) is misspecified, the analysis will still yield valid results.^40^ All models estimated school cluster–robust standard errors, accounting for clustering within children with repeated measures.^41^ Models also used heteroskedasticity weights by mean number of patients per school. Assumptions of this model include irreversibility of treatment (treated schools remain treated), no anticipation (no treatment effect prior to policy adoption), and conditional parallel trends (in the absence of treatment, the difference in outcomes between treated and control groups is constant over time, conditional on covariates).^36^ We used a prediction model to impute values for the 1% of observations missing ISP data used to determine CEP eligibility and inclusion criteria (eMethods in Supplement 1).

As a secondary analysis, we stratified by school level (elementary, middle, and high school). We assessed for selection bias in attendance of OCHIN clinics among CEP-participating schools and repeated analyses in further-restricted models including (1) dropping all schools with an estimated (vs actual) ISP value to assess whether results were robust to imputation and (2) limiting the sample to schools with at least 10 or 20 students on average per year, to confirm that results do not change with more restricted cutoffs. We also repeated analyses in a larger unbalanced sample of schools to ensure results remained similar. Finally, because obesity is both a hypothesized moderator and mediator, we did not adjust for obesity prevalence in our primary models but did in a sensitivity analysis. BMI, used to compute obesity prevalence, was calculated as weight in kilograms divided by height in meters squared using height and weight in the EHR. Obesity was defined as BMI at or above the 95th percentile for age and sex according to Centers for Disease Control and Prevention growth charts.^33^ Analyses were conducted using StataMP, version 18 (StataCorp LLC) and R, version 4.3.2 (R Project for Statistical Computing) from April 1, to July 5, 2024.

## Results

The sample included 1052 schools followed up from 2013-2014 through 2018-2019, matched to 155 778 patients. Table 1 displays characteristics of patients and schools overall and by year schools first adopted the policy.^33,42^ The weighted mean (SD) number of patients per school was 156 (170). The mean (SD) age was 10.9 (3.2) years (range, 4-18 years). The mean (SD) proportion of male patients was 0.50 (0.10). The mean (SD) proportions of patients based on race and ethnicity were as follows: 0.04 (0.08) Asian patients, 0.46 (0.33) Hispanic patients, 0.01 (0.03) patients of multiple races, 0.01 (0.02) Native Hawaiian or Other Pacific Islander patients, 0.13 (0.22) non-Hispanic Black patients, 0.25 (0.26) non-Hispanic White patients, and 0.09 (0.09) patients with unknown race and ethnicity. The mean (SD) proportion of patients with public health insurance was 0.85 (0.14), and the mean (SD) proportion with a BMI in the EHR corresponding to the definition of obesity was 0.19 (0.08).^33^ Of 1052 schools, 707 (67.2%) were elementary schools, 168 (16.0%) were middle schools, and 157 (14.9%) were high schools. The mean (SD) proportion of students eligible for free or reduced-price meals was 0.76 (0.15). Schools came from 12 US states, but 670 (63.7%) were located in California (n = 388) and Oregon (n = 282).

**Table 1.** Patient and School Characteristics by Year Schools Adopted the Community Eligibility Provision^a^

Characteristic	Mean (SD)							
	Overall	Years schools adopted CEP					
		Never	2014-2015	2015-2016	2016-2017	2017-2018	2018-2019
Patient characteristics							
No. of patients per school	155.9 (170.4)	187.5 (224.2)	123.2 (107.8)	79.0 (60.9)	207.1 (201.5)	223.7 (139.4)	126.6 (125.7)
Sex, proportion of patients							
Female	0.50 (0.10)	0.52 (0.10)	0.50 (0.10)	0.51 (0.13)	0.50 (0.07)	0.49 (0.06)	0.51 (0.10)
Male	0.50 (0.10)	0.48 (0.10)	0.50 (0.10)	0.49 (0.13)	0.50 (0.07)	0.51 (0.06)	0.49 (0.11)
Age, y	10.9 (3.2)	12.2 (3.5)	9.8 (2.7)	11.2 (3.0)	9.8 (2.1)	9.3 (1.9)	12.2 (3.4)
Race and ethnicity, proportion of patients							
American Indian or Alaska Native	0.004 (0.02)	0.01 (0.02)	0.004 (0.01)	0.01 (0.01)	0.001 (0.01)	0.001 (0.01)	0.003 (0.02)
Asian	0.04 (0.08)	0.06 (0.08)	0.04 (0.08)	0.03 (0.06)	0.01 (0.04)	0.02 (0.03)	0.03 (0.07)
Hispanic	0.46 (0.33)	0.32 (0.25)	0.54 (0.31)	0.29 (0.24)	0.25 (0.32)	0.80 (0.21)	0.67 (0.30)
Multiple	0.01 (0.03)	0.02 (0.03)	0.01 (0.03)	0.02 (0.02)	0.01 (0.02)	0.004 (0.01)	0.01 (0.02)
Native Hawaiian or Other Pacific Islander	0.01 (0.02)	0.01 (0.02)	0.01 (0.02)	0.01 (0.02)	0.002 (0.01)	0.002 (0.01)	0.004 (0.02)
Non-Hispanic Black	0.13 (0.22)	0.08 (0.14)	0.11 (0.18)	0.23 (0.34)	0.49 (0.30)	0.08 (0.18)	0.05 (0.13)
Non-Hispanic White	0.25 (0.26)	0.40 (0.27)	0.19 (0.21)	0.32 (0.28)	0.15 (0.17)	0.05 (0.06)	0.14 (0.22)
Unknown	0.09 (0.09)	0.10 (0.08)	0.10 (0.10)	0.09 (0.09)	0.08 (0.07)	0.05 (0.04)	0.09 (0.13)
Health insurance type, proportion of patients							
Public (eg, Medicaid)	0.85 (0.14)	0.78 (0.16)	0.89 (0.12)	0.81 (0.15)	0.86 (0.09)	0.96 (0.06)	0.90 (0.13)
Private	0.08 (0.10)	0.13 (0.13)	0.05 (0.07)	0.10 (0.10)	0.04 (0.05)	0.03 (0.03)	0.06 (0.09)
Uninsured	0.07 (0.09)	0.08 (0.10)	0.06 (0.08)	0.10 (0.10)	0.10 (0.08)	0.01 (0.05)	0.03 (0.10)
Proportion of patients with obesity^b^	0.19 (0.08)	0.18 (0.08)	0.20 (0.09)	0.17 (0.10)	0.19 (0.07)	0.21 (0.09)	0.21 (0.09)
School characteristics							
Total No. of schools	1052	351	405	57	72	29	138
School type, No. (%)							
Elementary	707 (67.2)	210 (59.8)	312 (77.0)	37 (64.9)	56 (77.8)	20 (69.0)	72 (52.2)
Middle	168 (16.0)	70 (19.9)	48 (11.9)	8 (14.0)	5 (6.9)	6 (20.7)	31 (22.5)
High	157 (14.9)	65 (18.5)	41 (10.1)	11 (19.3)	8 (11.1)	1 (3.5)	31 (22.5)
Other	20 (1.9)	6 (1.7)	4 (1.0)	1 (1.8)	3 (4.2)	2 (6.9)	4 (2.9)
No. of students	809.5 (631.1)	1007.0 (834.1)	614.1 (303.0)	604.5 (297.6)	574.6 (150.9)	680.5 (217.4)	1188.2 (809.4)
Proportion of students eligible for free or reduced-price meals	0.76 (0.15)	0.65 (0.12)	0.82 (0.11)	0.77 (0.15)	0.92 (0.11)	0.87 (0.07)	0.82 (0.11)
Social Deprivation Index score^c^	78.0 (19.1)	70.6 (20.2)	80.7 (17.8)	76.5 (18.2)	89.1 (13.7)	81.1 (18.3)	81.4 (15.8)
Rurality^d^	1.9 (1.4)	2.3 (1.8)	1.7 (1.2)	2.1 (1.7)	1.2 (0.6)	1.8 (0.5)	2.0 (1.3)
State, No. (%)							
California	388 (36.9)	107 (30.5)	137 (33.8)	9 (15.8)	16 (22.2)	19 (65.5)	100 (72.5)
Oregon	282 (26.8)	129 (36.8)	120 (29.6)	25 (43.9)	1 (1.4)	2 (6.9)	5 (3.6)
Texas	105 (10.0)	17 (4.8)	57 (14.1)	5 (8.8)	2 (2.8)	1 (3.5)	23 (16.7)
Indiana	64 (6.1)	22 (6.3)	26 (6.4)	6 (10.5)	2 (2.8)	0	8 (5.8)
Ohio	64 (6.1)	21 (6.0)	1 (0.3)	0	38 (52.8)	2 (6.9)	2 (1.5)
Massachusetts	60 (5.7)	5 (1.4)	46 (11.4)	0	9 (12.5)	0	0
Washington	46 (4.4)	40 (11.4)	3 (0.7)	3 (5.3)	0	0	0
Minnesota	22 (2.1)	3 (0.9)	2 (0.5)	9 (15.8)	3 (4.2)	5 (17.2)	0
North Carolina	10 (1.0)	0	9 (2.2)	0	1 (1.4)	0	0
Alaska	6 (0.6)	5 (1.4)	1 (0.3)	0	0	0	0
Montana	3 (0.3)	1 (0.3)	2 (0.5)	0	0	0	0
Wisconsin	2 (0.2)	1 (0.3)	1 (0.3)	0	0	0	0

^a^ Characteristics of 1052 schools eligible for the Community Eligibility Provision matched to 155 778 patients followed from school year 2013-2014 through 2018-2019. The mean (SD) values were weighted by the mean number of patients per school.

^b^ Obesity in children is defined as body mass index at or above the 95th percentile for age and sex according to Centers for Disease Control and Prevention growth charts.^33^

^c^ Social deprivation index score is a composite measure of area-level deprivation ranging from 1 to 100, with 100 corresponding to highest level of deprivation.^42^

^d^ The rurality measure is from the 2013 US Department of Agriculture rural-urban continuum; the continuum is a scale from 1 to 9, with 9 indicating most rural.

**Figure 1.**
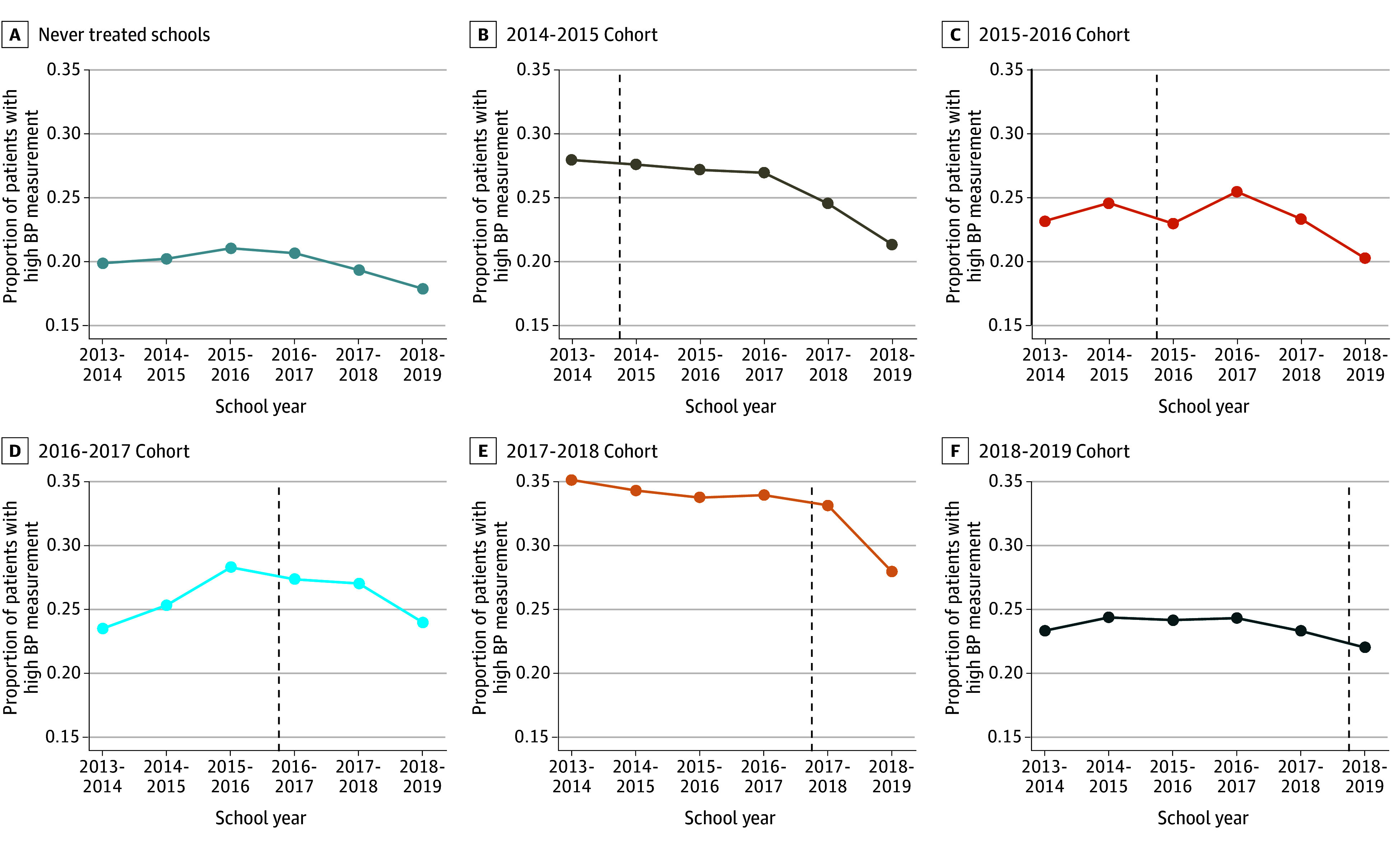
Unadjusted Trends in Proportion of Patients With a High Blood Pressure (BP) Measurement by Year Schools Adopted the Community Eligibility Provision Trends in yearly mean proportion of patients with a high BP measurement among 1052 schools followed from school year 2013-2014 through 2018-2019. BP measurements were from 155 778 unique patients. Mean values were weighted by the mean number of patients per school. Dashed lines indicate when the policy was first adopted by each cohort of schools. A high BP measurement is defined as systolic or diastolic BP at or above the 90th percentile for age, sex, and height based on children with normal weight (ie, body mass index below the 85th percentile for age and sex based on Centers for Disease Control and Prevention growth charts).^1^

Figure 1 displays unadjusted time trends in the weighted mean proportion of patients with a high BP measurement by cohort.^1^ The baseline proportion was 0.25 overall but varied by cohort. Table 2 shows results of the overall posttreatment average association of CEP and BP outcomes from the adjusted difference-in-differences models.^1,36^ By multiplying proportions by 100% to convert to percentage of patients with high BP, it was found that school CEP participation was associated with a −2.71 percentage point (pp) (95% CI, −5.10 to −0.31 pp; *P* = .03) net decrease in the percentage of patients with a high BP measurement. This corresponds to a −10.8% (95% CI, −20.4% to −1.2%) net decrease over 5 years. Participation in CEP was associated with a −2.48 pp (95% CI, −4.69 to −0.27 pp; *P* = .03) net reduction in the percentage of patients with a hypertensive measurement and a −2.21 pp (95% CI, −3.87 to −0.54 pp; *P* = .01) net reduction in the mean diastolic BP percentile. CEP was negatively associated with the mean systolic BP percentile, but the estimate was not statistically significant (point estimate, −0.50 pp [95% CI, −2.85 to 1.85 pp]; *P* = .68).

**Table 2.** Association of Participation in the Community Eligibility Provision With School-Level Blood Pressure Outcomes

Outcome	Difference-in-differences point estimate (95% CI)^a^
Primary outcome	
Patients with a high blood pressure measurement, %	−2.71 (−5.10 to −0.31)^b^
Secondary outcomes	
Patients with a hypertensive blood pressure measurement, %	−2.48 (−4.69 to −0.27)^b^
Mean diastolic blood pressure percentile	−2.21 (−3.87 to −0.54)^b^
Mean systolic blood pressure percentile	−0.50 (−2.85 to 1.85)

^a^ Estimates are expressed as percentage points. Sample includes 1052 schools matched to 155 778 patients followed from school year 2013-2014 through 2018-2019. High blood pressure measurement is defined as systolic or diastolic blood pressure at or above the 90th percentile for age, sex, and height based on children with normal weight (body mass index below the 85th percentile for age and sex based on Centers for Disease Control and Prevention growth charts); hypertensive blood pressure measurement is defined as a measurement at or above the 95th percentile.^1^ Treatment effects were estimated using a doubly robust difference-in-differences estimator described by Callaway and Sant’Anna,^36^ weighted by the mean number of patients per school, conditional on covariates.

^b^ *P* < .05.

**Figure 2.**
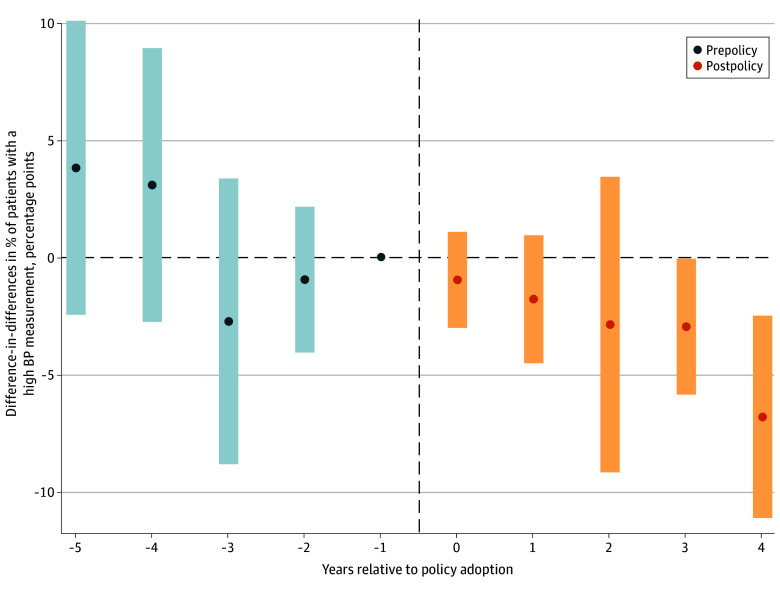
Difference-in-Differences Estimates of the Association of Participation in the Community Eligibility Provision With Percentage of Patients With a High Blood Pressure (BP) Measurement, Aggregated by Years Relative to Policy Adoption Sample includes 1052 schools matched to 155 778 patients followed from school year 2013-2014 through 2018-2019. A high BP measurement is defined as systolic or diastolic BP at or above the 90th percentile for age, sex, and height based on children with normal weight (ie, body mass index below the 85th percentile for age and sex based on Centers for Disease Control and Prevention growth charts).^1^ Prepolicy and postpolicy difference in differences were estimated using the doubly robust difference-in-differences estimator described by Callaway and Sant’Anna,^36^ weighted by mean number of patients per school, conditional on covariates. Point estimates and 95% CIs are shown. Estimates are aggregated by time since policy adoption (first year of policy adoption = 0; years prior to policy adoption are negative numbers, and years after policy adoption are positive numbers). The reference group includes eligible, nonparticipating schools (including both not-yet-participating and never-participating schools); reference year is 1 year prior to policy adoption (years relative to policy adoption = −1).

Figure 2 shows difference-in-differences estimates of the association of CEP and the percentage of patients with a high BP measurement by time since policy adoption.^1,36^ All postpolicy treatment effect estimates were negative, but only estimates for 4 and 5 years postpolicy were statistically significant. The joint test that prepolicy treatment effect estimates were different from zero was not statistically significant (χ^2^_10_ = 8.70; *P* = .56), lending support to the parallel trends and no anticipation assumptions. eFigure 2 in Supplement 1 displays pooled, postpolicy difference-in-differences estimates aggregated by cohort. Estimates were negative for all cohorts except for schools that adopted CEP in 2017-2018; however, only the estimate for the 2014-2015 cohort was statistically significant.

eTable 1 in Supplement 1 displays the results of stratified analyses by school level. The results were negative among all levels but only statistically significant among elementary schools. CEP was not significantly associated with the proportion of OCHIN patients in schools (eTable 2 in Supplement 1). The results of sensitivity analyses are displayed in eTable 3 in Supplement 1. The estimates and 95% CIs were similar when we dropped 11 schools with imputed ISP values. When we restricted the sample to schools with a mean number of 10 or more patients and 20 or more patients, the estimates remained negative but slightly attenuated. Results were similarly attenuated in an unbalanced sample with 556 additional schools and in a model adjusted for obesity prevalence.

## Discussion

Among 1052 schools followed from school year 2013-2014 through 2018-2019 matched to patients observed in community health organizations in several US states, participation in universal free meals via the CEP was associated with an 11% net decrease in the proportion of patients with a high BP measurement over a 5-year period. CEP was also associated with relative reductions in the proportion of patients with a hypertensive measurement and in the mean diastolic BP.

The observed association was strongest for the earliest cohort of schools, possibly because they had the longest exposure. Although CEP-participating schools were associated with larger reductions in high BP than eligible nonparticipating schools, trends in high BP decreased among both CEP-participating and nonparticipating schools. Although pediatric high BP prevalence increased in the US between 1988 and the early 2000s,^43,44^ it may now be decreasing.^2^ Future research should assess whether this decrease is associated with the timing of the Healthy, Hunger-Free Kids Act’s improved school meal nutrition standards.

In secondary analyses, CEP was associated with a net reduction in the proportion of patients with a high BP measurement in elementary schools specifically; this aligns with evidence that school meal participation is highest among elementary students.^45^ We were likely underpowered to detect an effect among middle and high schools; future research should prioritize assessing outcome differences by school category. In sensitivity analyses, results remained negative but were attenuated, and some estimates lost statistical significance when we restricted the sample to schools with higher numbers of patients. This is likely because excluding additional schools lowered our statistical power. However, changing the minimum number of patients from 10 to 20 did not further weaken the association, even though the sample size was restricted by approximately half.

Studies suggest that enhanced school meal nutrition standards were associated with reduced BMI and obesity risk for children from low-income families.^46,47^ These enhanced standards likely contribute to our observed results. Several studies have demonstrated that CEP is associated with relative reductions in obesity.^24,25,26^ This is consistent with our findings, as obesity can cause high BP.^1^ When we added obesity prevalence as a covariate in our models, associations were slightly diminished but remained negative, indicating that CEP may impact BP independently.

### Strengths and Limitations

The strengths of this study include its school-level longitudinal design, use of a comparison group of not-yet-participating and never-participating schools, and analytic approach that allowed treatment effects to vary over time and used a doubly robust estimation method to control for potential confounders. Additionally, evaluating pretreatment trends suggested that the parallel trends and no anticipation assumptions were reasonable.

This study has limitations. Data were not nationally representative; schools were concentrated predominantly in California and Oregon. However, the sample of patients from health clinics in low-income and racially and ethnically diverse areas represent a priority population for such interventions. We do not assume these findings to be representative for children from higher-income families; future research should assess this association in other settings and populations. Although CEP was associated with increased school meal participation,^22^ we cannot confirm that patients in the study participated in school meals. Excluding schools lacking data in all years decreased generalizability but increased internal validity. The proportion of patients with a high BP measurement observed in this study was higher than national estimates of high BP prevalence, likely because formal diagnoses require elevated measurements on 3 separate occasions.^1^ We acknowledge the possibilities of selection bias and confounding, despite our efforts to address them through analyses. Although our analytic approach controlled for all time-invariant unobserved school-level heterogeneity, it did not control for time-varying unobserved heterogeneity. To address concerns of selection bias, we showed that CEP was not associated with the proportion of students attending an OCHIN clinic. Nationwide, schools with OCHIN patients were also not independently associated with CEP adoption.^26^ Patients were matched to their closest neighborhood school based on address. There is likely to be some error in this assignment; however, we have no reason to believe school assignment differed by treatment status.

## Conclusions

In a cohort study school-level analysis of pediatric patients who visited community health organizations in 12 US states between 2013 and 2019, participation in the CEP was associated with a net decrease in the proportion of patients with a high BP measurement. The policy was also associated with a net decrease in the proportion of patients with a hypertensive measurement and in the mean diastolic BP percentile. These associations remained after adjusting for obesity prevalence. Universal free school meals policies such as the CEP represent promising strategies for improving population-level health.

## Notes

Supplement 1. **eFigure 1.** Flow Chart of Inclusion and Exclusion Criteria to Create Sample of Schools Matched to Patient Observations **eMethods.** Prediction Model to Impute Missing Values of School Identified Student Percentage (ISP) **eFigure 2.** Difference-in-Differences Estimates of the Association of Participation in the Community Eligibility Provision With Percent of Patients With a High Blood Pressure Measurement, Aggregated by Year of Policy Adoption **eTable 1.** Difference-in-Differences Estimates of the Association of Participation in the Community Eligibility Provision With Percent of Patients With a High Blood Pressure Measurement: Results of Analyses Stratified by School Level **eTable 2.** Association of Participation in the Community Eligibility Provision and Proportion of OCHIN Patients in Schools **eTable 3.** Association of Participation in the Community Eligibility Provision With School-Level Blood Pressure Outcomes: Results From Sensitivity Analyses
eReferences.

Supplement 2.
Data Sharing Statement
