# Supplementary material for: School Provision of Universal Free Meals and Blood Pressure Outcomes Among Youths
Source: JAMA Netw Open. 2025 Sep 25;8(9):e2533186. doi: 10.1001/jamanetworkopen.2025.33186 (PMC12464791; doi:10.1001/jamanetworkopen.2025.33186)
Supplement: Supplement 1. — eFigure 1. Flow Chart of Inclusion and Exclusion Criteria to Create Sample of Schools Matched to Patient Observations eMethods. Prediction Model to Impute Missing Values of School Identified Student Percentage (ISP) eFigure 2. Difference-in-Differences Estimates of the Association of Participation in the Community Eligibility Provision With Percent of Patients With a High Blood Pressure Measurement, Aggregated by Year of Policy Adoption eTable 1. Difference-in-Differences Estimates of the Association of Participation in the Community Eligibility Provision With Percent of Patients With a High Blood Pressure Measurement: Results of Analyses Stratified by School Level eTable 2. Association of Participation in the Community Eligibility Provision and Proportion of OCHIN Patients in Schools eTable 3. Association of Participation in the Community Eligibility Provision With School-Level Blood Pressure Outcomes: Results From Sensitivity Analyses eReferences. [file jamanetwopen-e2533186-s001.pdf]

Supplemental Online Content

Localio AM, Hebert PL, Knox MA, et al. School provision of universal free meals and blood pressure outcomes among youths. *JAMA Netw Open*. 2025;8(9):e2533186.
doi:10.1001/jamanetworkopen.2025.33186

eFigure 1. Flow Chart of Inclusion and Exclusion Criteria to Create Sample of Schools Matched to Patient Observations

eMethods. Prediction Model to Impute Missing Values of School Identified Student Percentage (ISP)

eFigure 2. Difference-in-Differences Estimates of the Association of Participation in the Community Eligibility Provision With Percent of Patients With a High Blood Pressure Measurement, Aggregated by Year of Policy Adoption

eTable 1. Difference-in-Differences Estimates of the Association of Participation in the Community Eligibility Provision with Percent of Patients With a High Blood Pressure Measurement**:** Results of Analyses Stratified by School Level

eTable 2. Association of Participation in the Community Eligibility Provision and Proportion of OCHIN Patients in Schools

eTable 3. Association of Participation in the Community Eligibility Provision With School-level Blood Pressure Outcomes: Results from Sensitivity Analyses

eReferences.

This supplemental material has been provided by the authors to give readers additional information about their work.

© 2025 Localio AM et al. *JAMA Network Open*.

eFigure 1. Flow Chart of Inclusion and Exclusion Criteria to Create Sample of Schools Matched to Patient Observations

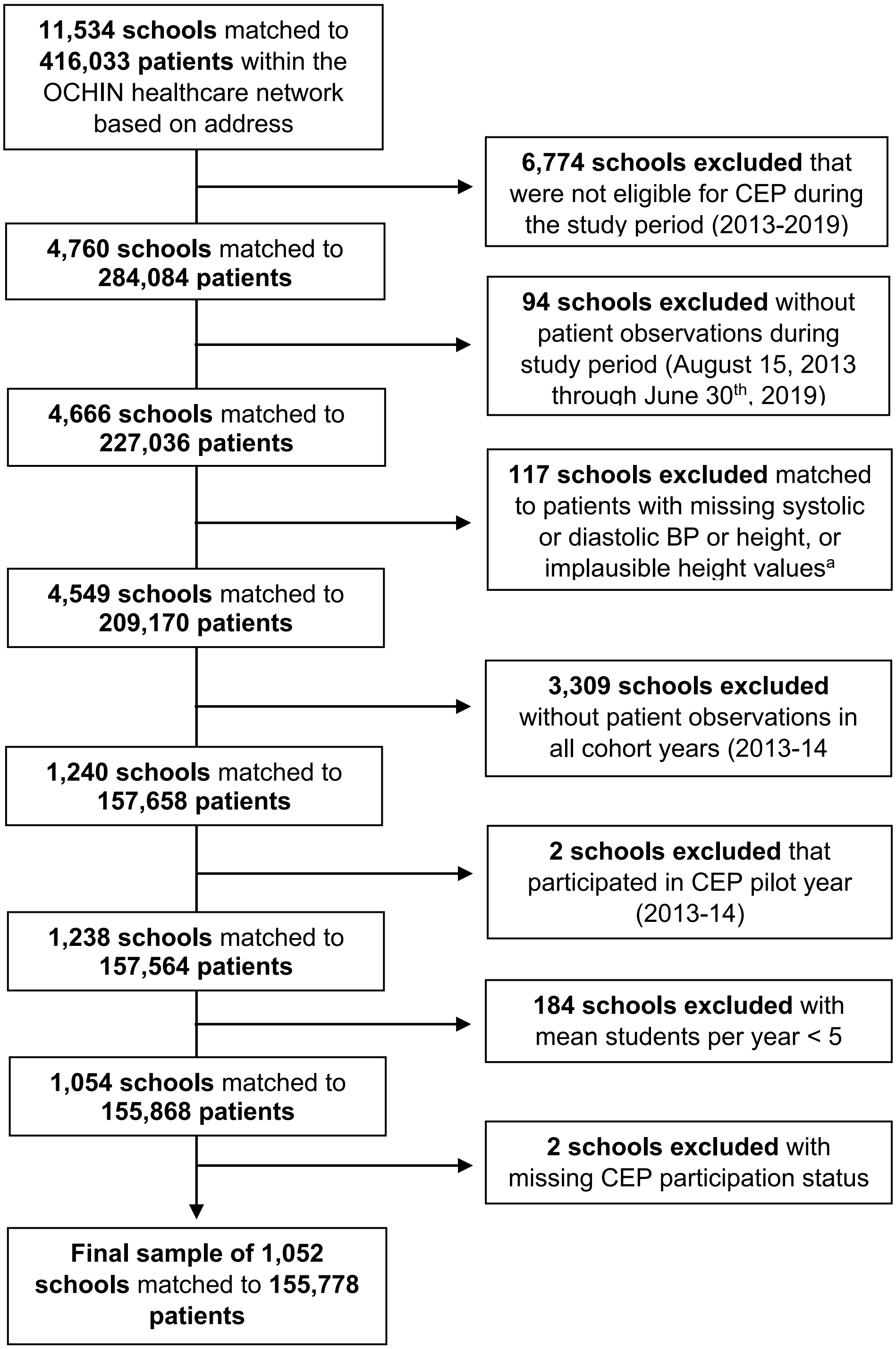

© 2025 Localio AM et al. *JAMA Network Open*.

[a]Implausible height values determined via two algorithms: one for cleaning child growth data from the electronic health record data,[1] and another for computing blood pressure percentiles based on age, sex, and height using normative blood pressure tables based on normal-weight children[2,3]

eMethods. Prediction Model to Impute Missing Values of School Identified Student Percentage (ISP)

The analysis included schools eligible to participate in CEP between 2014 (the first year CEP became available nationwide) and 2019. To be eligible for CEP, individual schools, groups of schools, or school districts must have an ISP of at least 40%. We used a school-level ISP of 40% or higher during the study period as a proxy for school CEP eligibility. To address the 1% of school ISP values that were missing, we used a prediction model to impute missing ISP values. We used an iteratively reweighted least squares logistic regression model to estimate ISP (ranging from 0 to 1) that included the following school-level covariates: a binary indicator for CEP participation, categorical variables for percent free or reduced-price meal eligible students, proportion Hispanic, Black, and White students, total number of students, district size, school type, and indicator variables for year and school state. We obtained predicted values of ISP for a random 20% of the sample and assessed the correlation between imputed ISP and real ISP values, which was 81%. We then reran the model using the entire dataset and predicted imputed ISP values. For those missing an ISP value, we replaced it with the imputed value. After obtaining a complete set of ISP values, we limited our sample to schools that had an ISP of 40% or higher during the study period.

© 2025 Localio AM et al. *JAMA Network Open*.

eFigure 2. Difference-in-Differences Estimates of the Association of Participation in the Community Eligibility Provision with Percent of Patients with a High Blood Pressure Measurement, Aggregated by Year of Policy Adoption

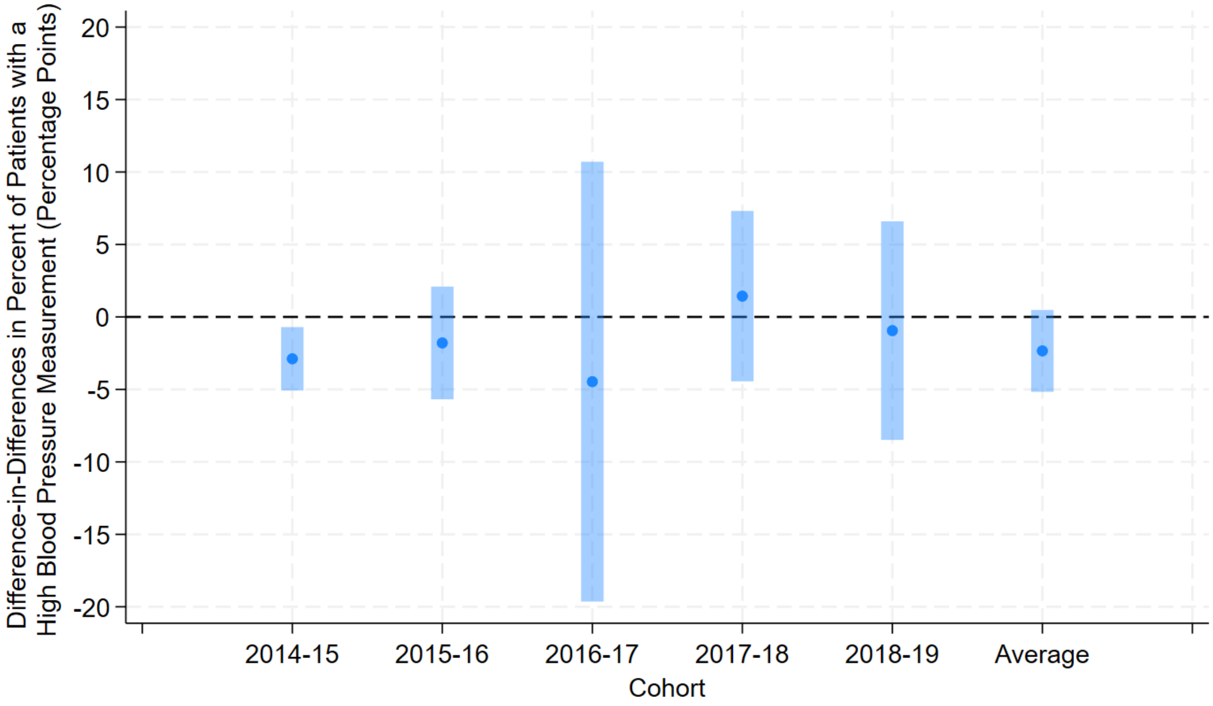

Sample includes 1,052 schools matched to 155,778 patients followed from school year 2013-14 through 2018-19. High blood pressure measurement is defined as systolic or diastolic blood pressure at or above the 90th percentile for age, sex, and height based on normal-weight children.[2] Treatment effects were estimated using Callaway/Sant'anna doubly robust difference-in-differences estimator,[4] weighted by mean number of patients per school, conditional on covariates. Reference group is eligible, non-participating schools (including both not-yet and never-participating schools); reference year is one year prior to policy adoption.

© 2025 Localio AM et al. *JAMA Network Open*.

eTable 1. Difference-in-Differences Estimates of the Association of Participation in the Community Eligibility Provision with Percent of Patients with a High Blood Pressure Measurement**:** Results of Analyses Stratified by School Level

	**N**	**Difference-in-Differences Point Estimate**	**95% Confidence Interval**
**School Level**			
Elementary	707	-3.00***	-4.84, -1.16
Middle	168	-0.16	-4.08, 3.76
High	157	-1.01	-4.40, 2.39

Analyses restricted to schools at each level followed from school year 2013-14 through 2018-19. 20 schools categorized as “other” (e.g. K-12) not included in analyses. Estimates expressed in percentage points. High blood pressure measurement is defined as systolic or diastolic blood pressure at or above the 90th percentile for age, sex, and height based on normal-weight children.[2] Treatment effects were estimated using Callaway/Sant’anna outcome regression estimator based on ordinary least squares,[4] weighted by mean number of patients per school, conditional on covariates.
*p<0.10 **p<0.05 ***p<0.01

eTable 2. Association of Participation in the Community Eligibility Provision and Proportion of OCHIN Patients in Schools

	**Point Estimate**	**95% Confidence Interval**	**p-value**
Difference-in-Differences in the Proportion of OCHIN Patients in Schools	2.08	-1.92, 6.08	0.31

Sample includes 1,052 schools followed from school year 2013-14 through 2018-19. Estimates expressed in percentage points. Outcome is defined as proportion of OCHIN patients out of total students in each school. Treatment effects were estimated using Callaway/Sant’anna outcome regression estimator,[4] weighted by mean number of patients per school.
*p<0.10 **p<0.05 ***p<0.01

© 2025 Localio AM et al. *JAMA Network Open*.

eTable 3. Association of Participation in the Community Eligibility Provision with School-level Blood Pressure Outcomes: Results from Sensitivity Analyses

	Difference-in-Differences Point Estimate	95% Confidence Interval
**Schools Missing Identified Student Percentage (ISP) Dropped from Sample[a]**		
Percent of Patients with a High Blood Pressure Measurement	-2.68**	-5.11, -0.24
Percent of Patients with a Hypertensive Blood Pressure Measurement	-2.45**	-4.70, -0.21
Mean Diastolic Blood Pressure Percentile	-2.24**	-3.95, -0.54
Mean Systolic Blood Pressure Percentile	-0.44	-2.82, 1.94
**Schools with Mean Students < 10 Dropped[b]**		
Percent of Patients with a High Blood Pressure Measurement	-1.93	-4.31, 0.45
Percent of Patients with a Hypertensive Blood Pressure Measurement	-1.52	-3.54, 0.51
Mean Diastolic Blood Pressure Percentile	-2.02***	-3.45, -0.60
Mean Systolic Blood Pressure Percentile	-0.57	-2.55, 1.40
**Schools with Mean Students < 20 Dropped[c]**		
Percent of Patients with a High Blood Pressure Measurement	-2.55**	-4.98, -0.11
Percent of Patients with a Hypertensive Blood Pressure Measurement	-1.90*	-3.83, 0.03
Mean Diastolic Blood Pressure Percentile	-1.74*	-3.72, 0.23
Mean Systolic Blood Pressure Percentile	-0.71	-3.09, 1.66
**Larger Unbalanced Sample of Schools[d]**		
Percent of Patients with a High Blood Pressure Measurement	-1.76	-3.87, 0.35
Percent of Patients with a Hypertensive Blood Pressure Measurement	-1.42	-3.35, 0.51
Mean Diastolic Blood Pressure Percentile	-1.54**	-2.85, -0.24
Mean Systolic Blood Pressure Percentile	-0.56	-2.24, 1.11
**Obesity Included as Covariate[e]**		
Percent of Patients with a High Blood Pressure Measurement	-1.99*	-4.09, 0.11
Percent of Patients with a Hypertensive Blood Pressure Measurement	-1.94	-4.43, 0.54
Mean Diastolic Blood Pressure Percentile	-1.78**	-3.19, -0.36
Mean Systolic Blood Pressure Percentile	-0.50	-2.42, 1.41

Estimates expressed as percentage points. High blood pressure measurement is defined as systolic or diastolic blood pressure at or above the 90th percentile for age, sex, and height based on normal-weight children; hypertensive blood pressure measurement is defined as a measurement at or above the 95th percentile.[2] Treatment effects were estimated using Callaway/Sant'anna doubly robust difference-in-differences estimator,[4] weighted by mean number of patients per school, conditional on covariates.
[a]Sample includes 1,041 schools followed from school year 2013-14 through 2018-19, that had non-missing identified student percentage (ISP) values in the original sample, before imputing missing values.
[b]Sample includes 828 schools followed from school year 2013-14 through 2018-19, that had non-missing identified student percentage (ISP) values in the original sample, before imputing missing values.
[c] Sample includes 595 schools followed from school year 2013-14 through 2018-19, that had non-missing identified student percentage (ISP) values in the original sample, before imputing missing values.

© 2025 Localio AM et al. *JAMA Network Open*.

[d]Sample included 1,608 schools that had at least two years of observations between 2013-14 and 2018-19, at least one “untreated” reference year, a mean number of patients of at least 5, and a non-missing CEP participation status
[e]Sample includes 1,052 schools followed from school year 2013-14 through 2018-19.
*p<0.10 **p<0.05 ***p<0.01

eReferences.

1. Daymont C, Ross ME, Localio AR, Fiks AG, Wasserman RC, Grundmeier RW. Automated identification of implausible values in growth data from pediatric electronic health records. *Journal of the American Medical Informatics Association*. 2017;24(6):1080-1087. doi:10.1093/jamia/ocx037

2. Flynn JT, Kaelber DC, Baker-Smith CM, et al. Clinical Practice Guideline for Screening and Management of High Blood Pressure in Children and Adolescents. *Pediatrics*. 2017;140(3):e20171904. doi:10.1542/peds.2017-1904

3. Rosner B, Cook N, Portman R, Daniels S, Falkner B. Determination of Blood Pressure Percentiles in Normal-Weight Children: Some Methodological Issues. *American Journal of Epidemiology*. 2008;167(6):653-666. doi:10.1093/aje/kwm348

4. Callaway, B, Sant’Anna, P. Difference-in-differences with multiple time periods. *Journal of Econonometrics*. 2021;225:200-230. doi:https://doi.org/10.1016/j.jeconom.2020.12.001

© 2025 Localio AM et al. *JAMA Network Open*.
