# Supplementary material for: School Provision of Universal Free Meals and Blood Pressure Outcomes Among Youths
Source: JAMA Netw Open. 2025 Sep 25;8(9):e2533186. doi: 10.1001/jamanetworkopen.2025.33186 (PMC12464791; doi:10.1001/jamanetworkopen.2025.33186)
Supplement: Supplement 2. — Data Sharing Statement [file jamanetwopen-e2533186-s002.pdf]

Data Sharing Statement

Localio. School Provision of Universal Free Meals and Blood Pressure Outcomes Among Youths. *JAMA Netw Open*. Published September 25, 2025. doi:10.1001/jamanetworkopen.2025.33186

Data
Data available: No

Additional Information
Explanation for why data not available: Data are from OCHIN healthcare Network
